# *Lactobacillus* and *Saccharomyces* fermentation products impact performance and the fecal microbiome in weanling pigs inoculated with enterotoxigenic *Escherichia coli*

**DOI:** 10.1093/jas/skae394

**Published:** 2025-01-22

**Authors:** Tanya Cherrington, David Jordan, John Pluske, Josie Mansfield, Kittitat Lugsomya, Stuart Wilkinson, David Cadogan, Sam Abraham, Mark O’Dea

**Affiliations:** Antimicrobial Resistance and Infectious Diseases Laboratory, Harry Butler Institute, Murdoch University, Murdoch, Western Australia, Australia; Antimicrobial Resistance and Infectious Diseases Laboratory, Harry Butler Institute, Murdoch University, Murdoch, Western Australia, Australia; Faculty of Science, The University of Melbourne, Parkville, Victoria, Australia; Australasian Pork Research Institute Ltd, Willaston, South Australia, Australia; Antimicrobial Resistance and Infectious Diseases Laboratory, Harry Butler Institute, Murdoch University, Murdoch, Western Australia, Australia; Antimicrobial Resistance and Infectious Diseases Laboratory, Harry Butler Institute, Murdoch University, Murdoch, Western Australia, Australia; Feedworks Pty Ltd, Romsey, Victoria, Australia; Feedworks Pty Ltd, Romsey, Victoria, Australia; Antimicrobial Resistance and Infectious Diseases Laboratory, Harry Butler Institute, Murdoch University, Murdoch, Western Australia, Australia; Antimicrobial Resistance and Infectious Diseases Laboratory, Harry Butler Institute, Murdoch University, Murdoch, Western Australia, Australia

**Keywords:** ETEC, pigs, liveweight, *Lactobacillus acidophilus* fermentation products (LFP), *Saccharomyces cerevisiae* fermentation products (SFP), microbiome

## Abstract

**Background:**

Enterotoxigenic F4 *Escherichia coli* (F4-ETEC) pose an economic threat to the swine industry through reduced growth, increased mortality and morbidity, and increased costs associated with treatment. Prevention and treatment of F4-ETEC often rely on antimicrobials; however, due to the threat of antimicrobial resistance, antimicrobial use is being minimized, and hence alternative control methods are needed. This study investigated the effects of postbiotics in the form of *Lactobacillus acidophilus* fermentation products (**LFP**) and *Saccharomyces cerevisiae* fermentation products (**SFP**), on pigs challenged with an F4 ETEC strain. Eighty pigs were selected based on a prescreening F4-ETEC susceptibility test. The animals were divided into 5 treatments each with 4 replicate pens. Pigs were assigned to 5 different diets: a control diet (CON); CON diet with 3,000 ppm ZnO (ZnO); CON diet with 2,000 ppm LFP (LFP); CON diet with 2,000 ppm SFP (SFP); CON diet with both 2,000 ppm LFP and 2,000 ppm SFP (LAS). Pigs were inoculated *per os* with F4-ETEC twice, on day 0 and day 1 of the experiment.

**Results:**

No significant differences in fecal consistency scores or fecal F4-ETEC concentration in pigs supplemented with LFP and/or SFP were detected. An increased diversity and abundance of *Lactobacillaceae* in the fecal microbiome of pigs supplemented with LFP were detected, as well as an increased final liveweight of pigs supplemented with LFP and/or SFP.

**Conclusion:**

This study demonstrated that the fecal microbiome is modified in F4-ETEC-challenged pigs supplemented with the combination of LFP and SFP, with these modifications previously associated with increased growth performance and health status in young pigs. Pigs receiving this combination of postbiotics also demonstrated an increased final liveweight, indicating that management of ETEC-associated performance loss may not require the complete removal of ETEC from a production system.

## Teaser

1. An increased diversity and abundance of *Lactobacillaceae* in the fecal microbiome of ETEC-challenged pigs supplemented with LFP were detected.

2. An increased final live weight of ETEC-challenged pigs supplemented with LFP and/or SFP was observed.

## Introduction

Newly weaned pigs are vulnerable to a multitude of infectious agents, with serotypes of enterotoxigenic *Escherichia coli* (**ETEC**) being amongst the most globally ubiquitous of these pathogens (Zimmerman et al., 2019). Infection with F4-ETEC can cause postweaning diarrhea (**PWD**), not only impacting the overall health of animals and potentially resulting in mortality but also incurring financial losses due to poor growth performance and inflated treatment and management costs (Fairbrother et al., 2005). Historically, antimicrobials have been used to control bacterial infections including ETEC. However, due to the emergence and dissemination of ETEC strains resistant to commonly used antimicrobials, coupled with regulations and industry initiatives to minimize antimicrobial usage, antimicrobials can no longer solely be relied upon for control (Jahanbakhsh et al., 2016; Laird et al., 2021).

Medicinal ZnO is a well-established feed additive and is recognized as a preventative option for F4-ETEC in the nursery. However, concerns with zinc pollution in the environment and heavy metal resistance co-selecting for antimicrobial resistance (Bednorz et al., 2013; Heo et al., 2013; Ciesinski et al., 2018) have resulted in the European Union banning veterinary products containing ZnO over than 150 ppm from June 2022 (EC, 2017). Numerous alternative in-feed and management-related strategies to ZnO including probiotics, postbiotics, bacteriophages, and vaccination programs are all being investigated for their efficacy and environmental impact in comparison to ZnO (Laird et al., 2021). Postbiotics, the fermentation products of probiotic strains, potentially offer an alternate strategy for minimizing antimicrobial use after weaning. The mixture of sugars, proteins, and amino acids in fermentation products is being investigated for advantageous effects on the gastrointestinal tract (**GIT**) microbiota and host health in protecting against pathogens and their associated diseases. *Lactobacilli* and the yeast species *Saccharomyces cerevisiae* are 2 researched probiotics, with studies describing alleviation in clinical signs of F4-ETEC infection in pigs (Lee et al., 2012; Trckova et al., 2014). The postbiotic potential of these species has been explored with studies reporting contrasting effects of *Saccharomyces cerevisiae* fermentation products (**SFP**) and *Lactobacillus acidophilus* fermentation products (**LFP**) against enteric pathogens in pigs (Kiarie et al., 2011, 2012; Brewer et al., 2014; Bass and Frank, 2017; Harris et al., 2017; Knapp et al., 2018). A reduction in ileal mucosa ETEC count and a reduction in the abundance of *Enterobacteriales* in the ileal microbiota has been reported in F4-ETEC-challenged pigs supplemented with SFP (Kiarie et al., 2011, 2012). No studies have investigated the effects of LFP on F4-ETEC-challenged pigs, with only 2 studies reporting effects in healthy weaner pigs, described as increased average daily gain (**ADG**), increased abundance of *Lactobacillus* and decreased abundance of *E. coli* (Lan et al., 2016; Bass and Frank, 2017).

These reported results suggest this postbiotic may potentially modulate the microbiome of F4-ETEC-infected pigs and minimize the effects of ETEC infection on growth performance. With weaning being the ideal target period for postbiotic treatment, due to piglets’ susceptibility to F4-ETEC infection and the potential to influence the rapid diversification of the GIT microbiota that occurs at this time (Guevarra et al., 2019), further investigation into these fermentation products is warranted. Using a controlled clinical trial with random allocation of pigs to treatment and blind assessment of outcomes, this study examined the impacts of LFP and SFP, administered alone or in combination, during and following F4-ETEC inoculation in pigs, and compared this to the effects of medicinal ZnO. It was hypothesized that these postbiotics would reduce the duration and severity of F4-ETEC infection, measured through the enumeration of fecal ETEC shedding and the presence of diarrhea. It was additionally hypothesized that LFP and SFP would improve growth performance and promote diversification of the microbiome.

## Materials and Methods

The experiment was conducted in an environmentally controlled room at Murdoch University. The experiment was conducted under protocols approved by the Animal Ethics Committee of Murdoch University, Murdoch, Western Australia (R3101/19) in accordance with the Australian code for the care and use of animals for scientific purposes (8th edition 2013).

### Animals, housing, and experimental design

Pigs received feed and water ad libitum. The LFP and SFP feed additives used in the trial were Diamond V SynGenX and Diamond V Original XPC, respectively. Pigs from a commercial piggery were weaned at 21 d of age and 80 of these, selected based on a prescreening F4-ETEC susceptibility test (Sterndale et al., 2019a), were transported to the animal housing facility at Murdoch University. Pigs were allocated into pens according to weaning weight (6.04 ± 1.07 kg (standard error of the mean, **SEM**)) and F4-ETEC susceptibility genetic testing with 4 pigs in each pen. Pens were constructed of metal with plastic flooring, and each contained a 5-space feeder, a nipple drinker, a manually filled water bowl containing electrolytes for the first 7 d after F4-ETEC challenge, and plastic bottles for enrichment. Pigs were acclimatized for 5 d upon arrival and housed at 28.0 ± 1.0 °C. The holding temperature was reduced to 26.0 ± 1.0 °C the day prior to, and on, both days of inoculation. The 5 treatment groups were allocated to pens by a randomized block design with 4 replicate pens of each treatment. The dietary treatments were i) control diet (CON), ii) CON diet supplemented with 3,000 ppm ZnO (ZnO), iii) CON diet supplemented with 2,000 ppm LFP (LFP), iv) CON diet supplemented with 2,000 ppm SFP (SFP), and v) CON diet supplemented with the combination of 2,000 ppm LFP and 2,000 ppm SFP (LAS). The base (CON) diet was manufactured by Specialty Feeds (Mundaring, Western Australia) and met the energy and nutrient requirements for pigs of this age (diet analysis is presented in Table S1).

### F4-ETEC inoculation

Following acclimation, pigs were inoculated with ETEC (serotype O149: F4: LT, STa, STb, EAST: β-hemolytic) on 2 consecutive days, designated as day 0 and day 1 (Table 1), according to the gelatin capsule method as described by Sterndale et al (2019b). Pigs received varying doses of F4-ETEC depending on the health of the pig since arrival at the facility with 2 pigs receiving 5.84 × 10^9^ colony forming units (**CFU**), 10 pigs receiving 9.2 × 10^9^ CFU, 5 pigs receiving 1.17 × 10^10^ CFU, 51 pigs receiving 1.5 × 10^10^ CFU, one pig receiving 1.84 × 10^10^ CFU All pigs received a full dose of 2 capsules on both days, unless showing diarrhea or lethargy, upon which they were assessed and received a half a dose of one capsule or no capsules. A total of 80 pigs were selected after MUC4 genetic prescreening, and of these 36 were fully susceptible, 37 partially susceptible, and 7 were non-susceptible. During the experiment pigs were removed from the study at different timepoints: due to death and welfare concerns, such as weight loss, poor health status and/or clinical diarrhea, a total of 11 pigs were excluded from the study. The final numbers in each group were: CON; *n* = 14, ZnO; *n* = 14, LFP; *n* = 14, SFP; *n* = 13, LAS; *n* = 14, as shown in Table S2.

**Table 1.** Outline of experimental design

Age (d)	Day in experiment	Trial Event
12	NA	Genetic prescreening
21	−5	Weaning and transport to facility
22 to 25	−4 ---> −1	Acclimation
26	0	First F4-ETEC dose
27	1	Second F4-ETEC dose
25 to 35	−1 ---> 9	Sample collection for microbiome analysis
26 to 33	0 ---> 7	Fecal sampling and processing for ETEC detection
27 to 33	1 ----> 7	RASP, ETEC and *E. coli* quantification
21 to 55	−5 ---> 28	Fecal shedding evaluation
28 to 55	−5 ---> 28	Production data collection for ADG, ADFI, FCR calculations

### Fecal sampling and processing

Rectal swabs were collected from all pigs on days 0, 1, 2, 3, and 7 postinoculation (pi). All swabs were streaked onto 5% sheep blood agar plates (Edwards Group, Australia) and incubated overnight at 37 °C. Plates were examined for colonies showing morphology consistent with *E. coli* and hemolysis representative of the challenge ETEC strain. Swabs were frozen at −20 °C for microbiome analysis. A single colony resembling the challenge ETEC strain was picked from each plate and inoculated into 500 μL of Luria Bertani broth in a 96-well format. These were grown overnight, and DNA was extracted using 6% chelex (Bio-Rad, Australia). To confirm the ETEC extracted was the challenge strain, a multiplex PCR using primers for the detection of fimbrial antigens K88 (F4), K99 (F5), 987 (F6), F41 and F18 and the enterotoxins STa, STb, and LTb and Shigatoxin Stx2e was performed (Casey and Bosworth, 2009). The PCR mix was prepared to a total volume of 15 µL consisting of 7.5 µL GoTaq Green Master Mix, 2 µL template DNA, and 0.5 µM of each primer and water. The thermocycling conditions were as described by Casey and Bosworth (2009). The products were run on a 2.5% gel at 80 V for 2 to 3 h and imaged on a BioRad Gel Doc (Life Science, California, USA).

### RASP ETEC and *E. coli* quantification

A total of 2 to 4 different fresh fecal samples were collected from pen floors on days 1, 2, 3, 4, and 7 pi and pooled by pen. The pooled pen fecal samples were processed using the Robotic Antimicrobial Susceptibility Platform (**RASP**) as outlined by Truswell et al. (2021) and Laird et al. (2022). Briefly, 1 g of feces was added to 19 mL of PBS buffer and placed in a stomacher machine (high setting) for 30 s. The contents were filtered upon pouring into sterile centrifuge tubes and then placed onto the RASP for dilutions and plating. Dilutions required for plating were estimated and then performed with 2 dilutions plated onto each agar plate. After overnight incubation at 37 °C, plates were placed back onto RASP for imaging and counting of colonies. The overall final dilutions were between 10^0^, 10^−1^, 10^−2^, 10^−3^, 10^−4^ from the original fecal concentration. Each sample was plated onto a 5% sheep blood agar plate and a Chromogenic ECC (MicroMedia, Australia) agar plate for quantification of putative ETEC and total *E. coli,* respectively. Sheep blood agar was selected for visual identification of ETEC colonies due to ETEC typically producing β-hemolysis when cultured on blood agar plates (Fairbrother et al., 2005).

Meanwhile, Chromogenic ECC agar plates are selective for *E. coli* with this species presenting as a blue colony. Images of plates were digitally captured and Pickolo software was calibrated to identify single colonies of ETEC and *E. coli* by image analysis based on color, hemolysis, size and circularity. Pickolo software (with manual assistance) was used to identify and count colonies, followed by species confirmation of representative *E. coli* colonies that were subjected to Microflex LT matrix-assisted laser desorption ionization-time of flight (MALDI-TOF) mass spectrometry analysis (Bruker, Germany), with data acquisition performed using flex control version 3.4. Mass spectra were compared against a reference library provided by the manufacturer via MBT Compass 4.1, and identification was based on the similarity scores derived from the mass profiles. A cutoff score of ≥ 2.0 was applied for reliable *E. coli* species-level identification. Colony forming units per gram (**CFU/g**) of feces were calculated from colony counts using SciRobotics software (Tecan, Switzerland) (Truswell et al 2021).

### Fecal consistency scores and pig performance evaluation

Feces were examined daily using a 5-point scale and scored as 1) dry and granulated; 2) dry and firm shaped; 3) moist and soft with retained shape; 4) pasty; or 5) watery diarrhea (Heo et al., 2009). Pigs were individually weighed weekly. Average daily feed intake (**ADFI**) at the pen level was calculated by dividing the total feed intake in pen by the total number of days on feed, ADG at the pen level was calculated by dividing the total weight gain in pen by the total number of days on feed and feed conversion ratio (**FCR**) at the pen level was calculated by dividing total feed intake in the pen by the total weight gain in pen. Pigs exited the trial facility 34 d after weaning.

### Microbiome diversity and abundance

The DNA was extracted from all rectal swabs using the MagMax DNA Multi-Sample Ultra kit (ThermoFisher Scientific, Australia) following the fecal samples protocol on a Kingfisher 96 particle processor (Life Technologies, USA). The V4 region of the 16S rRNA gene was amplified using the primers F515/R806 (Caporaso et al., 2011). Library preparation was performed using the Illumina 16S protocol per manufacturer’s instructions. Sequencing was performed on an Illumina Nextseq 500 platform using a 2 × 150 mid-output reagent kit. QIIME2 was used to process and analyze 16S rRNA gene sequencing data and to perform statistical calculations for alpha and beta diversity (Bolyen et al., 2019). Reads with a *q*-score greater than 30 were imported into QIIME2 for analysis using the Deblur pathway. Sequences were grouped into operational taxonomic units (OTUs) based on 97% sequence similarity using the Greengenes reference database. The OTUs were filtered and those with less than 10,000 reads (additive across samples) were removed from the dataset.

### Statistical analyses

Statistical analysis and graphing were conducted using STATA (v15.1 and v16.1), R Studio (v1.2.5033), and QIIME2 (Bolyen et al., 2019). Liveweight (kg) of pigs from days −5 to 27 (Table 1) was first analyzed descriptively to assess the form of temporal trends and generalized additive models were used to fit smoothing splines to nonlinear trends initially with pigs, pens and rooms as random effects in a full model. Simpler models were assessed for suitability based on the Akaike information criteria, and the final model was used to produce estimates of the mean effect of diet on liveweight through the experimental period with 95% confidence intervals relied on for interpreting the impact of sampling error on differences. A 1-way ANOVA followed by Tukey post hoc test was used to analyze performance data. Statistical significance was accepted at *P* < 0.05, and a trend was recognized at *P* < 0.1. Fecal consistency days, a measure of Fecal consistency scores (FCS) across time, were analyzed using a multilevel mixed-effects linear regression with pen as a random effect variable. Bacterial quantification was log-transformed with a multilevel mixed-effects linear regression used to analyze ETEC and total *E. coli* shedding across time, termed ETEC and ECC density, respectively. The abundance of bacterial families in the fecal microbiome was checked for normality and analyzed using the nonparametric Kruskal–Wallis H test. Post hoc comparisons were conducted with the Dunn’s test using Holm correction. Analysis of the microbiome data was conducted using Qiime2 with treatment groups compared to the control and ZnO diet to determine if feed additives altered the fecal microbiota. The nonparametric Kruskal–Wallis 1-way analysis of variance test was used to compare alpha diversity between treatment groups at each timepoint sampled (Xia and Sun, 2017). Faith’s phylogenetic diversity (Faith PD) was used to measure richness with Pielou’s evenness used to measure evenness (Hagerty et al., 2020). Differences in beta diversity of the fecal microbiome between treatment groups were analyzed across days using permutational multivariate analysis of variance (PERMANOVA). The PERMANOVA of the diversity analysis was calculated with the 999 Monte Carlo permutation and Benjamini-Hochberg correction (FDR) (Xia and Sun, 2017).

## Results

The *MUC4* genetic prescreening resulted in the selection of 36 fully susceptible, 37 partially susceptible, and 7 non-susceptible pigs for inclusion in the experiment due to high numbers of ETEC-F4 resistance in pigs. The ETEC PCR was conducted on a single colony from each ETEC-positive culture to determine presence of fimbrial antigens and enterotoxins. All colonies tested resembled the challenge strain through carriage of *lt, sta, stb,* and *k88* genes *astA* was present in the challenge strain but not included in the PCR, with no additional genes detected.

### Fecal consistency scores

**Figure 1.**
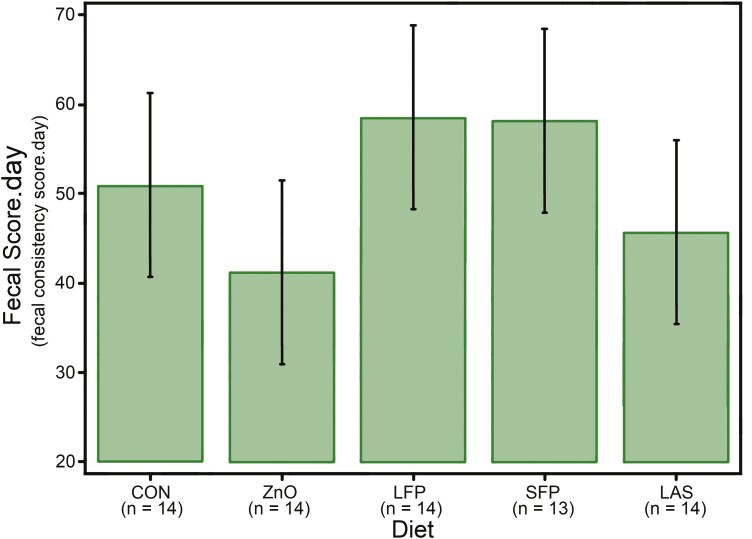
The mean fecal score days, a measurement relating to the fecal consistency score across the full duration of the study and the fecal score day.

Prior to ETEC inoculation, FCS were low across all dietary groups with 82% of FCS being 2 or less (Figure 1). This increased the day after inoculation with 75%, 99% and 60% of pigs having a FCS of 3 or above on days 1, 2 and 3, respectively. Scores of 4 or higher were present in 72% (*n* = 50/69) of pigs by day 2 with a FCS of 5, present in 20% of pigs.

The number of pigs with diarrhea remained high for approximately 4 d, dropping to a FCS of 2 in 85% of pigs on day 5. Analysis of fecal score days, a measure of FCS weighted by time, demonstrated no significant difference between diets when analyzed across the entire study (Figure 1) and when analyzed for the 4-d period after challenge, in which FCS increased.

### Fecal F4-ETEC concentrations

**Figure 2.**
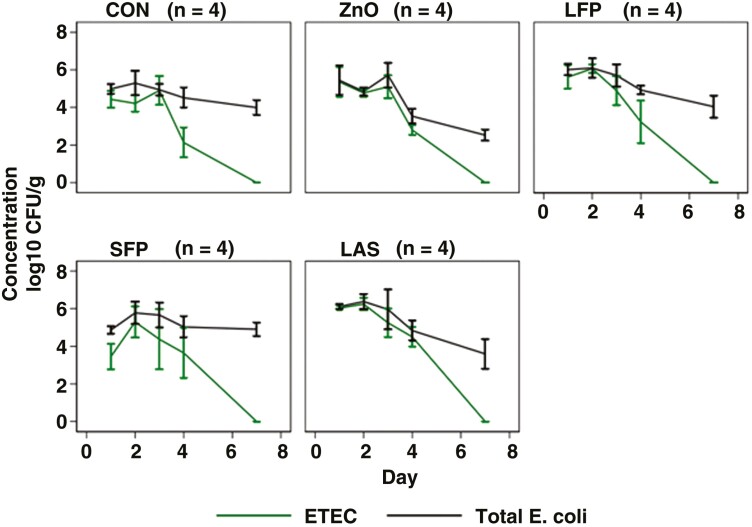
Quantification of fecal F4-ETEC and total putative *E. coli* in pigs belonging to different treatment groups following challenge with F4-ETEC. Error bars represent standard error of means. Pigs were inoculated with F4-ETEC on days 0 and 1.

**Figure 3.**
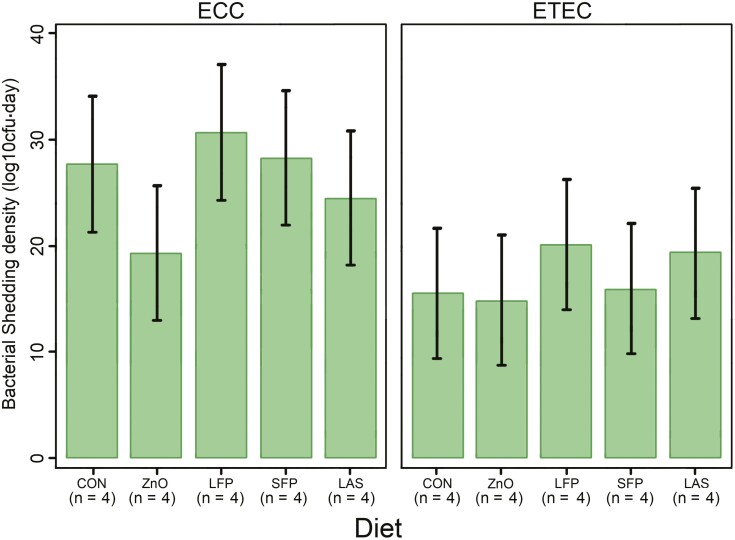
The mean F4-ETEC and total putative *E. coli* density. A measurement relating to the ETEC and putative total *E. coli* shedding across the 7 d following challenge with ETEC. Pigs were challenged with F4-ETEC on days 0 and 1.

Fecal F4-ETEC shedding was detected on days 1 to 4 pi, with ETEC shedding having cleared by day 7 in all groups (Figure 2). The average ETEC concentration across all pigs was 5.1, 5.3, 4.9, and 3.2 log_10_ CFU/g for days 1, 2, 3, and 4, respectively, showing a reduction in ETEC concentration over time (*P* < 0.05; 7.44e−13). Analysis of ETEC density [ETEC shedding across time (log CFU per g.d)] demonstrated no significant difference between diets with a shedding density of 15.6 (9.4, 21.8), 14.9 (8.7, 21.1), 20.2 (14.0, 26.4), 16.0 (9.8, 22.1), and 19.3 (13.1, 25.4) for the control, ZnO, LFP, SFP, and LAS groups, respectively (*P *= 0.431) (Figure 3). The total *E. coli* density also demonstrated no significant difference between diets (*P* = 0.675) (Figure 3).

Peak ETEC shedding differed amongst diets (Figure 2). Peak shedding was observed on day 1 for the ZnO group, day 2 for the LFP, SFP, and LAS groups, and day 3 for the control group. The percent of total *E. coli* comprised of ETEC was 92%, 94%, 87%, and 70% across days 1, 2, 3, and 4 supporting the initial increase in ETEC before clearance. This percentage of ETEC was most similar between groups on day 3, between 77% and 99%, but showed large variation on day 4 in which ETEC compromised 47% of all *E. coli* in the control group compared to 66%, 73%%, 79%, and 93% in the LFP, SFP, ZnO, and LAS groups, respectively.

### Pig performance

**Figure 4.**
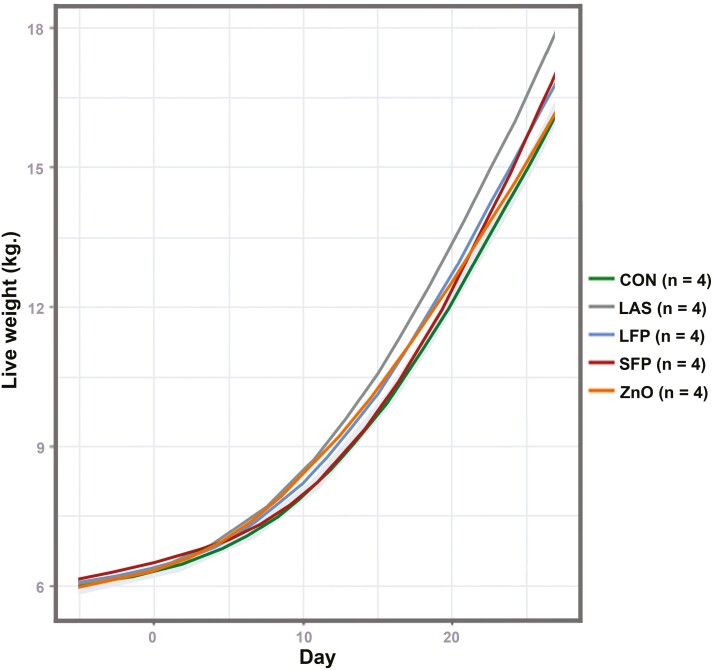
Mean effect of each of the 5 diets on liveweight after weaning over the duration of the trial. The effect was estimated by generalized additive models fitting smoothing splines to nonlinear changes in liveweight over time. Confidence intervals (shading) are provided for the each diet.

Starting weights were similar between all groups with a mean weight of 6.08 ± 0.13 (SEM) The mean liveweight (±95% CI) of pigs at the end of the study differed between diets and was highest in the LAS group at 17.9 kg (17.4, 18.3 kg) (Figure 4). In comparison, the ZnO and CON groups had the lowest mean live weight on the final day at 16.1 kg (15.8, 16.6) and 16.2 kg (15.9, 16.5) kg, respectively, approximately 1.6 kg lighter than pigs in the LAS group. On the final day of the study, the LFP and SFP groups had a mean liveweight of 16.9 kg (16.7, 17.0) and 17.0 kg (16.8, 17.2) kg, respectively.

ADG increased over time in all groups, with mean ADG in all pigs increasing from 60 g in week 1 to 208, 357, and 634 g in weeks 2, 3, and 4, respectively (Table 2). The ADG was highest in weeks 2, 3, and 4 for pigs in the LAS group, with the ADG being 95 and 140 g greater in the LAS group in Week 4 compared to the control and ZnO groups, respectively. Based on 1-way ANOVA, a statistically significant difference in ADG was noted between the CON and combined LAS diet (*P* = 0.021), but not between other diets at week 2 (*P *> 0.05). There was no significant difference in ADG at other timepoints (*P *> 0.05); however, the LAS diet showed a trend to significance (adjusted *P* = 0.051) when compared to ZnO at week 4 (Table 2).

**Table 2.** One-way ANOVA analysis of ADG, ADFI, and FCR, presented with their SEM, in pigs inoculated with F4-ETEC and fed different diets (*n* = experimental unit)

Item		Treatment				*P*-value
	CON (*n* = 4)	ZNO (*n* = 4)	LFP (*n* = 4)	SFP (*n* = 4)	LAS (*n* = 4)	
ADG (g)						
Week 1	63 ± 19.5	68 ± 11.8	59 ± 13.5	51 ± 22.7	60 ± 15.4	0.972
Week 2	169 ± 15.7^a^	233 ± 23.0 ^ab^	202 ± 19.9 ^ab^	187 ± 22.9 ^ab^	262 ± 31.8 ^b^	0.049
Week 3	348 ± 35.8	359 ± 33.1	380 ± 21.4	317 ± 38.1	380 ± 34.4	0.623
Week 4	602 ± 40.8	556 ± 37.9^*^	627 ± 17.2	690 ± 32.7	697 ± 40.3^*^	0.029
Overall	332 ± 73.4	312 ± 68.3	336 ± 43.2	334 ± 73.3	359 ± 87.0	0.387
ADFI (g)						
Week 1	94 ± 13.5	86 ± 8.8	92 ± 15.2	85 ± 14.8	90 ± 8.3	0.993
Week 2	255 ± 56.2	297 ± 28.8	271 ± 28.0	250 ± 52.4	290 ± 35.6	0.929
Week 3	454 ± 49.5	491 ± 39.9	488 ± 34.1	504 ± 71.3	484 ± 47.9	0.965
Week 4	872 ± 69.8	811 ± 19.0	893 ± 27.8	1028 ± 148.8	910 ± 97.9	0.567
Overall	422 ± 50.2	454 ± 61.3	482 ± 67.7	457 ± 67.02	481.81 ± 43.67	0.600
FCR (kg/kg)						
Week 1	2.13 ± 1.366	1.32 ± 0.237	1.58 ± 0.244	0.81 ± 1.419	1.55 ± 0.314	0.337
Week 2	1.38 ± 0.210	1.24 ± 0.087	1.32 ± 0.152	1.29 ± 0.273	1.27 ± 0.356	0.394
Week 3	1.31 ± 0.122	1.44 ± 0.153	1.27 ± 0.081	1.68 ± 0.374	1.27 ± 0.147	0.090
Week 4	1.45 ± 0.088	1.45 ± 0.051	1.42 ± 0.058	1.47 ± 0.279	1.34 ± 0.190	0.733
Overall	1.50 ± 0.107	1.41 ± 0.044	1.39 ± 0.028	1.50 ± 0.348	1.32 ± 0.167	0.601

^a,b^Mean values within a row that have different superscripts are significantly different (*P *< 0.05).

^*^ *P*-value = 0.051 following Tukey post hoc test.

ADFI increased over time with mean ADFI in all groups starting between 86 and 94 g in Week 1 and increasing to between 811 and 1,028 g in the final week. The SFP group had the highest ADFI in week 4 (1,028 g) with all other groups having an ADFI between 811 and 910 g; however, there was no statistically significant difference between groups at any timepoint (*P* > 0.05). The FCR demonstrated the greatest variation in Week 1 (days −5 to 2) ranging from an average of 0.8 in the SFP group to 2.1 in the control group. The average FCR across treatment groups was less variant at other timepoints, and no significant differences were detected (*P* > 0.05).

### Pig fecal microbiome

**Figure 5.**
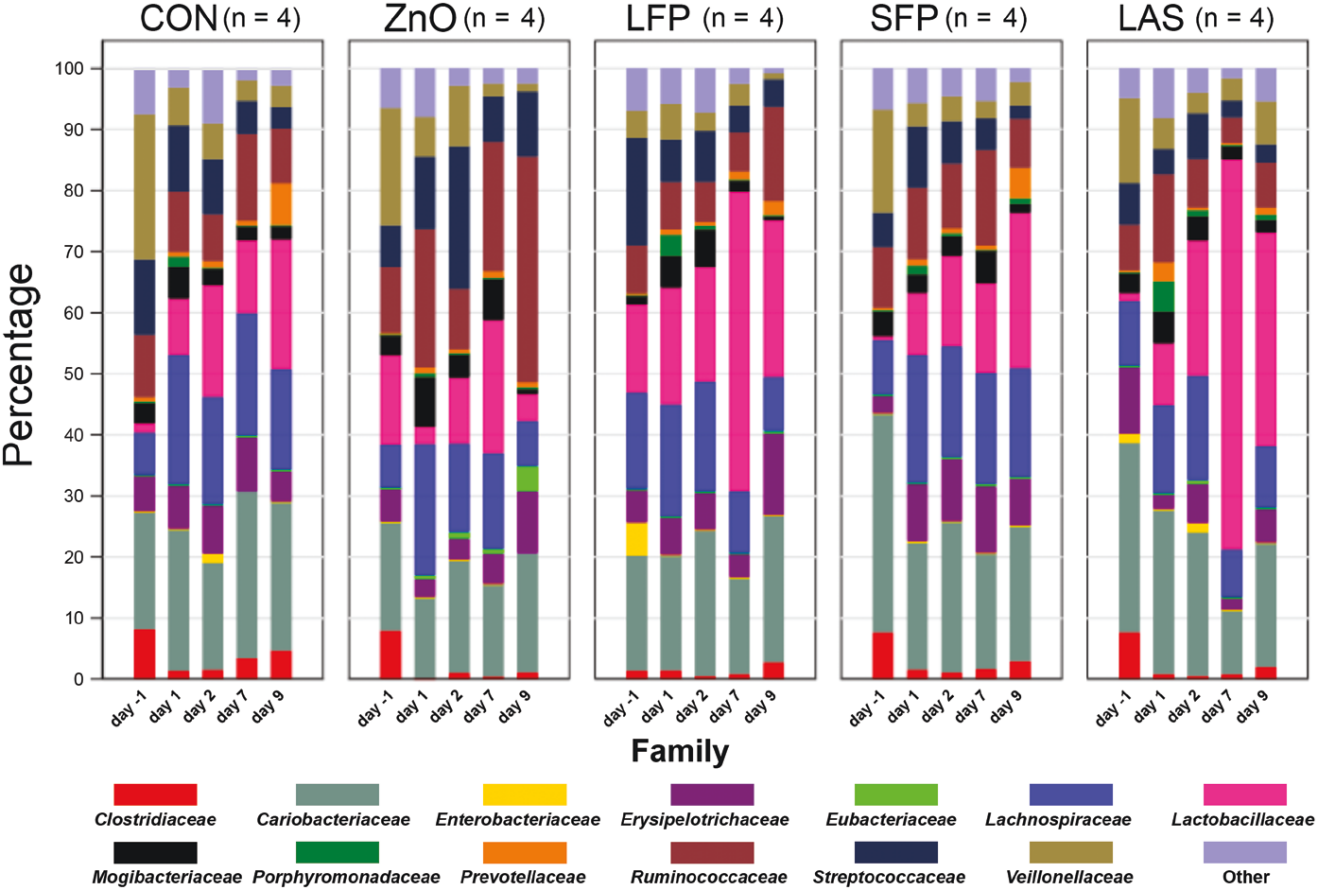
Proportion of bacterial families in fecal microbiota of F4-ETEC-challenged pigs over multiple days and treatment groups.

Analysis of the fecal microbiota identified the dominant bacterial families across all groups to be *Coriobactericaceae*, *Lactobacillaceae*, *Lachnospiraceae*, and *Ruminococcaceae* (Figure 5). The abundance of the 2 major bacterial families, *Lactobacillaceae* and *Ruminococcaceae*, differed between treatment groups on day 7. *Lactobacillaceae* abundance was increased in the LFP (*P* = 0.030) and LAS (*P* = 0.003) diets compared to the CON group. Further analysis of the *Lactobacillaceae* detected all OTU’s identified to the genus level to belong to *Lactobacillus* and 6 different species composing this family. The dominant species across all treatment groups was *Lactobacillus reuteri* composing 50.5%, 64.3%, 72.9%, 76.9%, and 81.2% for the ZnO, LFP, SFP, control, and LAS groups, respectively. No species were identified in greater proportion between diets. Meanwhile, the ZnO group had an increased abundance of *Ruminococcaceae* compared to the LFP (*P* = 0.023) and LAS (*P* = 0.026) groups. The 2 dominant species within this family were identified to be *Faecalibacterium prausnitzi* and *Ruminococcus bromii*. However, no difference in percent abundance of these species was detected between these treatment groups.

On day 1 pi, the abundance of *Clostridiaceae* increased (*P* = 0.017) in the ZnO group compared to the SFP group. This was attributed to the significantly increased proportion of the genus *SMB53* and reduction in the genus *Sarcina* in the ZnO group compared to the SFP group. There was no difference (*P* > 0.05) in the proportion of *Enterobacteriaceae* between treatment groups at any timepoint.

Analysis of alpha diversity detected a greater richness, measured using Faith’s PD index, in the LAS group compared to CON on day 1 pi and compared to the ZnO group when all timepoints were analyzed together. The significant results are shown in Table 3. Meanwhile, the SFP group had greater richness than the ZnO group on day 2 (*P* = 0.038). No significant difference in evenness, measured using Pielou’s evenness index, was detected between diets at each timepoint analyzed (*P* > 0.05). The beta diversity was analyzed using 3 indices: Jaccard distance, unweighted UniFrac, and weighted UniFrac, with analysis comparing treatment groups to the control and ZnO groups. The significant results are shown in Table 4. On day 1 and day 7, the ZnO group demonstrated a difference in microbial composition compared to diet CON (*p*_ZnO.CON.day1_* *= 0.020 and *p*_ZnO.CON.day7_ = 0.020). Differences in microbial composition were also detected between the ZnO group and the LFP and SFP groups on day 7 (*P* = 0.047 and *P* = 0.020, respectively). No differences in the microbial composition of treatments compared to the control group were detected on days −1, 2, or 9 (*P *> 0.05). Overall, the ZnO group differed to all other groups when all sample timepoints were analyzed together (*p*_ZnO.CON_ = 0.007, *p*_ZnO.SFP_ = 0.005, *p*_ZnO.LFP_ = 0.048, and *p*_ZnO.LAS_ = 0.005). All of the data for microbiome diversity for analyses are shown in Table S3.

**Table 3.** Pairwise comparison (Kruskal–Wallis test) of Faith’s phylogenetic diversity index of treatment groups in F4-ETEC-challenged pigs with significantly different alpha diversity, when compared to the control and ZnO groups across timepoints (*n* = experimental unit)

Day	Group 1 ^*^	Group 2	Faith’s PD	
			*H*	*P*-value
Overall	LAS (*n* = 4)	ZnO (*n* = 4)	4.138	0.042
1	LAS (*n* = 4)	CON (*n* = 4)	5.014	0.025
2	SFP (*n* = 4)	ZnO (*n* = 4)	4.321	0.038

^*^Group 1 is the treatment with greater alpha diversity detected.

**Table 4.** Pairwise comparisons (PERMANOVA test) of beta diversity indices of treatment groups in F4-ETEC-challenged pigs with significantly different beta diversity, when compared to the control and ZnO group across timepoints (*n* = experimental unit)

Day	Group 1	Group 2	Unweighted UniFrac	Jaccard distance	Weighted UniFrac
			*q*	*q*	*q*
Overall	CON (*n* = 4)	ZnO (*n* = 4)	0.010	0.007	0.083
	ZnO (*n* = 4)	SFP (*n* = 4)	0.010	0.005	0.070
	ZnO (*n* = 4)	LFP (*n* = 4)	0.218	0.048	0.180
	ZnO (*n* = 4)	LAS (*n* = 4)	0.010	0.005	0.180
1	CON (*n* = 4)	ZnO (*n* = 4)	0.067	0.020	0.477
7	CON (*n* = 4)	ZnO (*n* = 4)	0.010	0.020	0.126
	ZnO (*n* = 4)	LFP (*n* = 4)	0.273	0.047	0.217
	ZnO (*n* = 4)	SFP (*n* = 4)	0.195	0.020	0.131

## Discussion

The necessity to reduce the use of both antibiotics and ZnO in the feed of newly weaned pigs demands the development of novel strategies to negate the impact of bacterial disease in this species. In this study, we evaluated 2 microbial-derived products, separately and in combination, as postbiotics for amelioration of F4-ETEC infection. The current study demonstrated no significant effects of LFP, SFP, and their combination, on FCS, and the duration and quantity of F4-ETEC fecal shedding in F4-ETEC-challenged pigs, when compared to a standard diet or the established ZnO intervention, although numerical trends for improvements were evident especially with the combination of products. However, the large variation between pigs in the number of fecal score days caused the lack of statistical significance.

No previous studies have reported the effects of LFP or the combination of postbiotics in F4-ETEC-challenged pigs. However, results from the current study support data from Kiarie et al. (2011) reporting no effect of SFP on FCS and fecal ETEC concentration in F4-ETEC-challenged pigs. Meanwhile, a reduction of F4-ETEC was observed in ileal mucosa scrapings of pigs supplemented with SFP (Kiarie et al., 2011). Kiarie et al. (2011) also reported no effect of SFP, whether supplemented in-feed or in drinking water, on ADG in ETEC-challenged pigs. These data contrast with the current study, with pigs supplemented with LFP and SFP having an increased final liveweight compared to the control and ZnO groups. Furthermore, the combined effect of these 2 products demonstrated a trend towards statistical significance when compared to ZnO, as opposed to single product use, with a final liveweight of 17.9 kg (17.4, 18.3). The discrepancies in the impacts of SFP between these studies may be due to the ETEC inoculation methods and prescreening selection of susceptibility. Although no direct effect on ETEC infection was detected in the parameters measured, the increased growth performance suggests the feed additives may indirectly alleviate ETEC infection through variations to the fecal microbiota. Identification and implementation of feed additives that manipulate the microbiome, improving defence against pathogens, may lead to a reduced reliance on antimicrobials in the future.

The present study showed that the abundance of *Enterobacteriaceae* in the fecal microbiota was similar between all dietary groups. Nevertheless, numerous changes to the fecal microbiota following F4-ETEC-challenged were identified when pigs received feed-supplemented with LFP (LFP group), and LFP and SFP (LAS group). Bacterial diversity was increased in pigs receiving the combination of the LFP and SFP compared to the control and ZnO groups. Furthermore, the abundance of *Lactobacillaceae* was increased in pigs receiving feed-supplemented LFP, whether alone or in combination with SFP, compared to the control and ZnO groups. An increased bacterial diversity and increased abundance of *Lactobacillaceae* have previously been demonstrated to be associated with increased health status and growth performance (Dou et al., 2017; Ober et al., 2017; Lu et al., 2018; Guevarra et al., 2019). The dominant species comprising this family was *L. reuteri*, with the probiotic effect of *L. reuteri* in the swine GIT well documented. Studies have demonstrated its production of antimicrobial substances including reuterin and reutericyclin and its strong ability to adhere to and colonize the GIT (Hou et al., 2015). This adhesion is pivotal for probiotic effects including pathogen exclusion and immune modulation. Furthermore, supplementation with *L. reuteri* has been reported to enhance the intestinal mucosal barrier and immune stimulation in newborn pigs by increasing villous height, the crypt depth of the jejunum, the number of goblet and CD3^+^ T cells, and the expression of antimicrobial peptides, IL-4 and IFN-γ (Wang et al., 2020). Improved growth performance was also reported in these nursery pigs whilst other studies have reported increased growth performance in pigs supplemented with *L. reuteri* (Hou et al., 2015; Wang et al., 2020). While no reduction in ETEC shedding or FCS were reported in the current study, the direct effects of the postbiotics and the effect of the increased proportion of Lactobacillaceae in the small and large intestine may account for the increased weight in these pigs. However, we were unable to measure these factors in this study.

An increased abundance of *Ruminococcaceae* was detected in pigs supplemented with ZnO compared to the LFP and LAS groups in the current study. This family has previously been detected at a higher abundance in healthy pigs compared to diarrheic pigs (Dou et al., 2017). *Ruminococcaceae* have been reported at high levels within the cecum with *F. prausnitzi* associated with fiber fermentation and butyrate production (Benus et al., 2010). Fermentation of fibers in the large intestine lowers the pH value, with acidic conditions reducing the growth of pathogenic bacterial strains including ETEC (Heo et al., 2013). Despite the increased abundance of this family and species, no variation in ETEC fecal shedding density or FCS was detected between treatments in the current study. Meanwhile, the functional role of the bacterial genera SMB53, detected at high levels in pigs supplemented with ZnO in the current study, remains unclear. However, it has previously been reported as a dominant genus within the small intestine of weaner pigs (Pollock et al., 2021).

Overall, the alteration of the microbiome in pigs supplemented with the combination of both LFP and SFP, detected as an increased alpha diversity and increased abundance of *Lactobacillaceae*, may account for their increased liveweight. Whilst understanding of the microbiome requires further expansion, these postbiotics demonstrate potential to alleviate the reduced growth performance imposed by F4-ETEC infections following weaning. In this regard, biologically worthwhile investigations into controlling ETEC-associated PWD need to be performed in vivo. Traditionally, monitoring shedding and disease following an F4-ETEC infection has been achieved by assessing FCS, or by laboratory-based culturing of F4-ETEC scored or described as pure, mixed, or no ETEC growth. Both methods show low resolution and due to the subjective scoring nature, lack comparability between studies. Recently, Luise et al. (2019) provided a highly detailed review of ETEC models, suggesting the use of both clinical and ETEC-specific biomarkers for the analysis of ETEC infection. The current study followed these suggestions, recording ETEC infection through enumeration of fecal ETEC shedding and the presence of diarrhea through FCS. Bacterial quantification offers accurate and comparable data, removing subjectivity from traditional bacterial scoring methods. However, this method can be highly laborious and, being time sensitive, can be unachievable for large sample numbers during these already labor-intensive in vivo models. Implementation of high throughput robotic platforms, such as the RASP, can greatly reduce the labor costs associated with bacterial quantification. Accurate measurement of ETEC infection through methods including ETEC quantification has potential benefits in assessing the effects of alternative ETEC control strategies in in vivo models, and as such, this method warrants use against other traditional methods with further analysis.

In conclusion, this study demonstrated that the fecal microbiome is modified in F4-ETEC-challenged weaner pigs supplemented with the combination of LFP and SFP, with these modifications previously associated with increased growth performance and health status in pigs. Pigs receiving this combination of postbiotics also demonstrated an increased final liveweight, indicating that management of F4-ETEC-associated performance loss may not require the complete removal of ETEC from a production system.

## Supplementary Material

skae394_suppl_Supplementary_Table_S1

skae394_suppl_Supplementary_Table_S2

skae394_suppl_Supplementary_Table_S3

## Abbreviations

ADFI: average daily feed intake
ADG: average daily gain
CON: control diet
ETEC: enterotoxigenic *Escherichia coli*
F4-ETEC: F4**-**enterotoxigenic Escherichia *coli*
Faith PD: Faith’s phylogenetic diversity
FCR: feed conversion ratio
FCS: fecal consistency scores
GIT: gastrointestinal tract
LAS: control diets with 2,000 ppm LFP and 2,000 ppm SFP
LFP: *Lactobacillus acidophilus* fermentation products and control diet with 2,000 ppm *Lactobacillus acidophilus* fermentation products
MALDI-TOF: Matrix-assisted laser desorption ionization-time of flight
PERMANOVA: permutational multivariate analysis of variance
PWD: postweaning diarrhea
RASP: Robotic Antimicrobial Susceptibility Platform
SEM: standard error of the mean
SFP: *Saccharomyces cerevisiae* fermentation products and control diet with 2,000 ppm *Saccharomyces cerevisiae* fermentation products
ZnO: control diets with 2,000 ppm ZnO
