# Supplementary material for: Lactobacillus and Saccharomyces fermentation products impact performance and the fecal microbiome in weanling pigs inoculated with enterotoxigenic Escherichia coli
Source: J Anim Sci. 2025 Jan 22;103:skae394. doi: 10.1093/jas/skae394 (PMC11842899; doi:10.1093/jas/skae394)
Supplement: skae394_suppl_Supplementary_Table_S1 [file skae394_suppl_supplementary_table_s1.docx]

**Supplementary table 1** Analyses of diet components used in the experiment

| Parameter | Units | CON | ZnO | LFP | SFP | LAS |
| --- | --- | --- | --- | --- | --- | --- |
| Ash | g/100g | 5.8 | 5.8 | 5.6 | 5.7 | 5.7 |
| Crude Fibre | % | 1.9 | 1.9 | 2.1 | 2 | 1.8 |
| Fat, Total | g/100g | 5.8 | 5.3 | 5.4 | 4.7 | 4.8 |
| Metabolisable Energy (calculated) | MJ/kg | 13.40 | 13.09 | 13.11 | 13.02 | 13.0 |
| Moisture | g/100g | 9.1 | 10.5 | 10.5 | 10.1 | 10.8 |
| Nitrogen-Free Extract | % | 57.4 | 55.9 | 55.9 | 57.1 | 56.4 |
| Protein | g/100g | 20 | 20.6 | 20.5 | 20.4 | 20.5 |
| Standardised ileal digestible Lysine (calculated) | % | 1.23 | 1.23 | 1.23 | 1.23 | 1.23 |
| Available phosphorus (calculated) | % | 0.5 | 0.5 | 0.5 | 0.5 | 0.5 |
| Calcium (calculated) | % | 0.8 | 0.8 | 0.8 | 0.8 | 0.8 |
