# Supplementary material for: Lactobacillus and Saccharomyces fermentation products impact performance and the fecal microbiome in weanling pigs inoculated with enterotoxigenic Escherichia coli
Source: J Anim Sci. 2025 Jan 22;103:skae394. doi: 10.1093/jas/skae394 (PMC11842899; doi:10.1093/jas/skae394)
Supplement: skae394_suppl_Supplementary_Table_S2 [file skae394_suppl_supplementary_table_s2.docx]

| Seq | group | numbers of pigs in each pen | total pigs | total pens |
| --- | --- | --- | --- | --- |
| 1 | CON | 3, 4, 4, 3 | 14 | 4 |
| 2 | ZnO | 4, 3, 3, 4 | 14 | 4 |
| 3 | LFP | 3, 3, 4, 4 | 14 | 4 |
| 4 | SFP | 4, 2, 4, 3 | 13 | 4 |
| 5 | LAS | 4, 3, 3, 4 | 14 | 4 |
| summary | | | 69 | 20 |
